# The Outbreak of the COVID-19 Pandemic and its Social Impact on Education: Were Engineering Teachers Ready to Teach Online?

**DOI:** 10.3390/ijerph18042127

**Published:** 2021-02-22

**Authors:** Víctor Revilla-Cuesta, Marta Skaf, Juan Manuel Varona, Vanesa Ortega-López

**Affiliations:** 1 Department of Civil Engineering, University of Burgos, 09001 Burgos, Spain; vrevilla@ubu.es (V.R.-C.); vortega@ubu.es (V.O.-L.); 2 Department of Construction, University of Burgos, 09001 Burgos, Spain; 3 Department of Organizational Engineering, University of Burgos, 09001 Burgos, Spain; jmvarona@ubu.es

**Keywords:** COVID-19 pandemic, social science, social activities, human behaviors, empathy, face-to-face teaching, online teaching, engineering courses, student’s perception, teacher support

## Abstract

The major impacts of the COVID-19 pandemic are still affecting all social dimensions. Its specific impact on education is extensive and quite evident in the adaptation from Face-to-Face (F2F) teaching to online methodologies throughout the first wave of the pandemic and the strict rules on lockdown. As lesson formats changed radically, the relevance of evaluating student on-line learning processes in university degrees throughout this period became clear. For this purpose, the perceptions of engineering students towards five specific course units forming part of engineering degree courses at the University of Burgos, Spain, were evaluated to assess the quality of the online teaching they received. Comparisons were also drawn with their perceptions of the F2F teaching of the course units prior to the outbreak of the pandemic. According to the students’ perceptions, the teachers possessed the technical knowledge, the social skills, and the personal capabilities (empathy and understanding of the at times troubled situation of each student) for a very abrupt adaptation of their courses to an online methodology. The shortcomings of the online teaching were related to its particularities and each teacher’s personality traits. Overall, engineering teachers appeared well prepared for a situation of these characteristics and, if similar online teaching scenarios were ever repeated, the quality of engineering teaching appears to be guaranteed.

## 1. Introduction

Humanity is currently facing a completely new, unexpected, and extremely serious situation: the COVID-19 pandemic. Undoubtedly, the most dramatic situation occurred at the outset, catching both the governments and the populations of so many countries unprepared [1]. The imposition of a strict lockdown in some countries lessened the impact of the first wave of the pandemic, although with intense economic damage and harm to both social and personal networks [2]. Nevertheless, the virus continues to infect and daily reports of new infections, hospital bed occupancy rates, and higher numbers of deaths are now incessant. When early detection is insufficient, almost all countries across the globe are adopting measures to reduce the spread of the virus [3], such as local lockdowns in areas with high infection rates [4]. However, lockdown and its associated measures on commercial and social activity reduce purchasing power and impoverish the population [5], in addition to its serious effects on social behavior [6]. The great challenge of managing this pandemic is therefore to find the right balance between health, social, and economic dimensions; no easy task when any improvement in one generally worsens the others [7]. Continuous learning and responsible behavior are the only ways to ensure that these measures are as effective as possible in the three abovementioned aspects [8], and so that the negative impacts in various social dimensions are minimized, such as in university teaching, which is the focus of this article [6].

The social consequences of the current coronavirus situation are numerous and varied [6]. Without any doubt, the foremost is the social isolation of the population during confinement [9], although any subsequent changes to the way we are expected to interrelate are also extremely relevant, due to social distancing obligations [10] and limits on gatherings of family members and at social events [11]. These restrictions have even caused significant change in the workplace (companies, offices …) and at educational centers (schools, universities …), among others, including circulation areas and healthcare corridors, and restrictions on the use of available spaces [12]. Nevertheless, children have probably been the most vulnerable under these circumstances, as the initial confinement and social distancing pressures limit their personal development through play, the activity through which their first interpersonal relationships often begin [13]. Access to professional fields has also seen a sudden increase in remote telework [14]; graduates entering employment are now expected to substitute personal interaction for tele-conferencing [15]. In addition, workers in other fields have seen their professional careers interrupted and anxiety over their professional future is increasing as a result of the associated economic crisis [16]. Finally, a change in the most sought-after university careers has even been observed [17], in so far as students appear to be leaning towards courses of a social nature [18].

In addition to the abovementioned areas, these changes to the way people are expected to interact have also significantly affected many other areas of great social relevance, such as education, culture, and research [19]. The effects on education become especially relevant, as education equips new generations with the necessary knowledge to contribute to social development and welfare [20]. The need to adapt Face-to-Face (F2F) teaching to online teaching is still a great challenge for both teachers and students [21], due to the stark contrast between both teaching methods [22]: while in F2F teaching, the pace of work is moderated whenever the student calls on the teacher to facilitate an understanding of the concepts [23], in online teaching, students must self-regulate their activity and understand a greater number of concepts alone [24]. Online teaching requires a very detailed and careful preparation by the teacher, because it is the only way to guarantee proper student learning [25]. In addition, to achieve successful learning, students must progressively develop their capacity for self-regulation, defining their own study schedules [26], which have to be perceived as an obligation to become a habit [27]. However, the first wave of the COVID-19 pandemic, the consequent lockdown, and the interruption of F2F classes meant that teaching had suddenly to be adapted to online methodologies [28] for which neither teachers nor students were prepared [29]. This situation might imply that concepts were not learnt as well in online classes as they had been in F2F classes, with the inevitable conclusion that the teaching quality was worse [30]. If it were to persist, it might imply poorly trained students with negative consequences for society [31]. Added to this complex situation, the psychological malaise of both confinement [32] and isolation from close family and friends [33], such as stress [34], anxiety [35], sleep problems [36], and feelings of fear [37], must also be considered. Online teaching has therefore to be adapted to an even more complicated scenario [38], in order to avoid serious consequences [39]: teachers had to consider the personal situation of the students when defining the level of demand, or the online teaching method (synchronous, asynchronous …) [9].

Both teachers and students have faced this sort of teaching situation and its negative impacts, which appear to be most alarming in university education [12]. Final-year university students soon to complete their education and to embark upon a career may not have acquired certain knowledge, due to the interruption of classes and the switch to online teaching, which will never be formally taught to them [40]. Moreover, in some fields, such as public health and psychiatry, the effects of COVID-19 have ratcheted up both productivity and pressure at work, with no standard process of adaptation and learning at work for recent graduates [41]. Nevertheless, teaching staff have to use this situation for improving the quality of teaching and learning [42], as lockdown has taught us valuable lessons [43]. Firstly, the great importance of study sites for social education, which cannot be performed online, has been asserted. The habits and behaviors that are acquired at all stages of learning, from elementary to university education, will define people’s social behavior [44]. Secondly, lockdown has demonstrated the need to manage student emotions to improve learning. The teacher has not only to transmit the concepts, but has also to foster a learning environment in which the concepts explained are also easily learned [45]. Finally, many teachers have first-hand experience of new technologies and are aware of their utility for approaching certain aspects that are often overlooked in traditional teaching, such as creativity and a critical spirit, so they will be very likely, in the future, to incorporate these tools more often in their classes [46]. In short, these experiences can be used in all fields to improve the quality of their teaching.

### Aim and Scope

The adaptation of F2F teaching to online teaching is easier in classes where theoretical learning prevails [22]. However, its adaptation is more complex in fields such as University Engineering Degrees, where the practical application of theoretical concepts assumes great relevance [47]. It is mainly due to the greater difficulty of explaining practical concepts online, because of the absence of direct contact between teacher and student [48]. Therefore, the unexpected adaptation to online teaching of engineering courses as a consequence of the COVID-19 pandemic could lead to significantly worse learning patterns among students. This situation could have great social relevance regarding the contribution of engineering to the development of society [49].

The aim of this paper was to analyze the quality of teaching in the field of engineering studies during the lockdown caused by the first wave of the COVID-19 pandemic. This study intended to determine whether the learning of theoretical and practical concepts among students was successful; if the teachers could adapt the teaching material to online teaching with sufficient clarity and quality; if the teachers were available to dispel the doubts of the students in effective ways; and if the students thought that the teacher was concerned about their learning and personal situation. Finally, the aim was also to understand the overall assessment of the students regarding the teaching quality received during the confinement. In this way, it may be ascertained whether the training of engineering students was acceptable for their future professional life.

To do so, the perceptions among students of both F2F and online teaching imparted on five university engineering course units forming part of two Bachelor’s Degrees and one Master’s Degree of the University of Burgos, Spain, were evaluated. As all the aspects were analyzed from the point of view of the students, this study may be considered as an evaluation of the teacher’s work and their behavior during teaching. Moreover, the selected course units covered almost all subject matter taught on the engineering degrees and the teachers were likewise selected as the most representative. In this way, relevant conclusions may be drawn for the improvement of teaching in both modalities on these university degree courses, so that quality teaching practice among engineering teachers may be reinforced.

## 2. Materials and Methods

### 2.1. Experimental Design: Framework and General Marks

The suspension of classes at the University of Burgos, Spain, due to the first wave of the COVID-19 pandemic, took effect on Thursday, 12 March 2020 at 3:00 pm. At that time, six weeks of F2F teaching of the second semester had been imparted. Three days later, on Sunday, 15 March 2020, a strict lockdown was imposed in Spain, interrupting all F2F classes at both university and non-university levels. During the following week, the University communicated to all teachers and students that teaching would be online until the end of the academic year (15 May 2020), including the final evaluation exams (June 2020), regardless of the evolution of the COVID-19 pandemic. This was a measure aimed at trying to normalize an already extremely exceptional situation.

The classes at the University of Burgos were suspended on a Thursday and the experiment had been designed before the following Monday, so that it started on the first week of online teaching. During those four days (from Thursday to Sunday, both inclusive), different actions were taken:

- The guidelines that the teachers of the course units selected for the experiment had to follow during the online teaching were established, so that the online teaching was comparable between teachers [50]. These guidelines were of a general nature and each teacher had to adapt to them on the basis of their own knowledge and past experience. The implementation of the experiment was therefore very close to reality [22]. These guidelines are listed in Section 2.2.
- Course units of different types were selected for the experiment, so that they were of a general character [49]. In addition, course units with similar teacher profiles were selected, in order to compare the perceptions of online teaching and F2F teaching [48]. Both these aspects and the process of selecting the course units are explained in detail in Section 2.3.
- The evaluative survey was prepared. This survey regarding F2F teaching was administered to the students on the selected course units in the first week of online classes. At the end of the experiment (last week of online classes), the students responded to the survey on online teaching. In this way, students’ perceptions of both teaching methodologies and a variety of other aspects could be compared. The questions in this survey are listed in Section 2.5.

At the end of the experiment, the answers of the students from the selected course units during both F2F and online teaching were subjected to a statistical analysis, a qualitative analysis, and a mixed analysis based on word counting. The type of analysis performed depended on the type of question. This analysis is explained in detail in Section 2.6.

### 2.2. Online Teaching Guidelines

During the few days available for the experimental design, the guidelines that the participating teachers had to follow during the online teaching were defined by the authors of this research work. These guidelines were of a general nature, so that the teachers had some freedom to adapt them in accordance with their own knowledge and past experience [48]. However, it was essential to ensure that all the teachers followed them, in order to ensure comparable results [50]. Consideration was given to the authors’ knowledge of the characteristics of online teaching and the need to balance family life with teacher and student commitments for the definition of the guidelines.

It was established that the online teaching would be asynchronous, which implied that the teacher would never demand that students were available or connected at a certain time to a particular online platform, such as Skype or Microsoft Teams [51]. Teaching was therefore flexible and adapted to the different personal and family situations of both the teachers and the students in such an exceptional situation. The use of audio or video recordings was recommended for teaching, in which both theoretical and practical concepts were explained.

The teachers were also asked to maintain contact with their students through periodic messaging to inform them of new course content and other relevant topics. In addition, they were expected to respond to all issues that the students raised as quickly as possible via email, so that the learning of course content could be as immediate as possible. Finally, the teachers were asked to establish a tutorial schedule, listing availability and a web tool (Skype, Microsoft Teams, Zoom, Blackboard Collaborate …). In this way, students could discuss doubts that might otherwise be too complex to be solved through multiple email messages. The tutorial schedule, once defined, had to be communicated to students during the first week of online teaching. These actions were intended to support students, because their learning throughout the course unit in asynchronous online teaching depends largely on their motivation and interest [51], which will be greater if they feel that the teacher cares about them and is actively involved in their learning [52].

Finally, it is important to emphasize that no guidelines were established for teachers on how to grade the course units (exams, individual or group projects …). Teachers had to grade the course units according to the teaching guides, since these are settled at the beginning of the course.

### 2.3. Selected Course Units

The second step was to select the course units for the experimental study. If the experiment was to be both relevant and widely representative, three fundamental criteria had to be fulfilled:

- Course units of almost all types from engineering education had to be included.
- The profile of the teacher on each selected course unit had to fit an almost standard one: a specialized teacher with regard to the content of the course unit, with knowledge of new technologies and capable of developing course unit activities online [53]. In addition, the F2F teaching methodology of the teachers had to be similar, so that any effect of the teacher on the comparison between F2F and online teaching could be disregarded [52].
- Students of as many ages as possible should participate.

The course units for the experiment were selected according to the criteria set out above through a sequential process, avoiding an excessive amount of data that might otherwise have hindered the analysis:

1. Firstly, all course units from all four years of the Bachelor’s Degree in Agroalimentary Engineering & the Rural Environment, the Bachelor’s Degree in Civil Engineering, and the Master’s Degree in Civil Engineering of the University of Burgos were identified. These three university degrees were chosen in representation of all areas of the Higher Polytechnic School of the University of Burgos, with regard to both type of knowledge taught and teaching levels (Bachelor’s Degree and Master’s Degree). Moreover, the authors of this study had formed working relationships of partnership and trust with most of the teachers of these careers, which facilitated their involvement in the experiment.
2. Secondly, all the courses were divided into three different groups, according to the type of knowledge that the students are expected to acquire. On the one hand are the Basic Course Units (BCU) that have no engineering-related content, on which concepts of a general nature are taught, such as mathematics, applied sciences and economics. On the other hand are the Design Course Units (DCU) on which technical concepts are taught, such as structural, hydraulic, and thermal theory, as well as computing design. Finally, the Management Course Units (MCU) were selected, on which the future engineers are expected to learn the necessary concepts for professional practice unrelated to engineering design, such as employee management, project timelines, and budgeting. These groups of course units are widely accepted as essential aspects for the training of engineers [47,49].
3. In each of these three groups, four course units were selected, which approximately covered the age range of students, from 18 to 24 years old. These course units were selected on the basis of the teachers’ profiles. Their profiles had to fit the one indicated above and the participating teachers had to teach their F2F classes in a similar way, considering such aspects as time dedicated to both theoretical and practical concepts, class methodology, group work, and workload of the qualification. In this way, the students’ perceptions of both F2F and online teaching could be compared on all the selected course units [52]. A comparative process between the available teachers was conducted, to select suitable course units, based on the prior knowledge of the authors, the evaluation surveys from previous years on teaching activity administered to students, and the teaching quality evaluation grade obtained by each teacher of the University of Burgos (DOCENTIA program [54]).
4. Finally, the teachers of the selected course units (four in each group, twelve course units in total) were contacted to explain the experiment, its objectives, and what they were expected to do. Agreement was forthcoming from the teachers of five different course units (1 BCU, 2 DCU, and 2 MCU)—a response that was considered acceptable, in view of the social impacts of the COVID-19 pandemic (family and professional life balance, confinement, teleworking …) that had recently begun to make themselves felt in Spain [30].

In view of the sequential design, the five selected courses covered the three previously indicated types of courses (BCU, DCU, and MCU) and the teachers’ profiles were all similar. Moreover, students of a wide range of ages had enrolled in the course units (see Section 2.4), so the perceptions of students of different levels of maturity and experience could be analyzed. The five course units were:

- Business Economics II, a BCU on the 2nd year of the Bachelor’s Degree in Agroalimentary Engineering & the Rural Environment.
- Construction & Agroalimentary Building, a DCU on the 3rd year of the Bachelor’s Degree in Agroalimentary Engineering & the Rural Environment.
- Engineering of Green Spaces, a DCU on the 4th year of the Bachelor’s Degree in Agroalimentary Engineering & the Rural Environment.
- Project and Construction Management, an MCU on the 3rd year of the Bachelor’s Degree in Civil Engineering.
- Engineering Projects, an MCU on the 1st year of the Master’s Degree in Civil Engineering.

### 2.4. Participants

The average age and the number of students following each of the above course units are shown in Table 1. In addition, these data are also shown for the different groups of course units (BCU, DCU, and MCU). Overall, the median age of the 66 participating students was 21.59 ± 2.47 years old. No distinction was made between either the gender of the participants or whether they were repeating the course unit, since the aim was to establish their perceptions towards the online teaching they had received from a general point of view, rather than for specific population groups [10]. On the other hand, as indicated above, the age range covered in the study was wide, which combined with the considerable number of participating students and provided the study with a high level of representativeness. All the university course units in the study are usually taught F2F. Therefore, this situation offered the first online teaching experience for all participating students, which meant that the students were more demanding regarding the teacher’s work [22].

On the other hand, the 6 participating teachers had a mean age of 43.82 ± 11.39 years. All of them had a Master’s degree in engineering (3 agricultural engineers, 2 civil engineers, and 1 industrial engineer) and none of them had ever taught online throughout their teaching career. However, 3 of them reported that they had attended courses on the general aspects of successful online teaching.

### 2.5. Instrument: Survey

The last step was to design the survey to be administered to the students. The survey had to elicit student perceptions [47], while collecting clear information for analysis to facilitate the interpretation of the results [55]. Therefore, a numerical valuation survey was considered the best option. In this survey, the students were asked to express their agreement or otherwise with a series of statements on a 5-point Likert-type scale (1, strongly disagree; 2, disagree; 3, unsure; 4, agree; 5, strongly agree). However, the general viewpoints behind their perceptions were also considered of interest, as aspects that were not addressed in the numerical-answer questions of the survey could be detected [55]. An open question was therefore also included.

The survey consisted of 21 statements/questions and addressed five different areas, each of high relevance for quality teaching. These areas were defined after analyzing other similar research works [22,50,56,57,58]: explanation and learning of theoretical concepts (statements 1–3), explanation and learning of concepts for practical application or exercises (statements 4–6), quality of teaching material (statements 7–9), teacher’s availability to communicate with students and the channels of communication in use (statements 10–12 and question 13), and teacher’s attitude during teaching (statements 14–16). Finally, students were asked about their overall perception of the course (statements 17–20 and question 21). The statements/questions included in the survey were as follows:

1. The theoretical concepts have been properly explained.
2. I would be able to face a real problem related to the course work with the theoretical knowledge that I have learnt.
3. It was easy for me to understand the theoretical concepts explained in class.
4. The practical concepts have been properly explained.
5. My practical knowledge will be sufficient to deal with a real problem related to the course unit.
6. It was easy for me to understand the practical concepts explained in class.
7. The material provided was prepared for the type of teaching received.
8. The teaching material had been carefully prepared in the right format.
9. All material provided was necessary for a proper understanding of the course unit.
10. It was easy to enter into contact with the teacher and to clarify doubts.
11. The teacher responded to all doubts that were raised regardless of their nature, even if so-called “silly questions” were asked.
12. The teacher quickly responded to the doubts as they were raised.
13. If you communicated with the teacher at some point, how did you do so (F2F, email, Skype, Microsoft Teams …)? The answer to this question was analyzed as a qualitative variable (see Section 2.6).
14. I felt that the teacher was concerned that students would understand the concepts that had been explained.
15. The teacher continuously monitored the understanding of the concepts that had been explained, for example, by asking whether students had understood them.
16. Teachers showed sympathetic attitudes towards project assignment completion dates, mainly when handed in late.
17. The course was difficult to understand.
18. The course was difficult to pass.
19. I have learned a lot during the course.
20. The course required a lot of work.
21. If you think that they exist, indicate the advantages/disadvantages of the online teaching compared to the F2F teaching or vice versa. What shortcomings if any did you associate with each teaching methodology?

During the design process of the survey, both the validity and the reliability of the instrument (survey) were analyzed. The validity was evaluated by performing a confirmatory factorial analysis, which showed that the six categories of questions considered in the survey explained 86.8% of the variance. The reliability was evaluated by conducting a Cronbach’s Alpha test, for which a value of 0.788 was obtained. The values were considered acceptable for the use of the questionnaire.

As mentioned in Section 2.1, the students following the selected course units were administered the survey in the first and in the last week of online classes. Their responses during the first and the last week respectively revealed their perceptions both of the F2F teaching and of the online teaching that they had received. In this way, it was possible to compare the students’ perceptions of each teaching typology between each course group (BCU, DCU, and MCU) and in a global way (all courses together). Question 21 (open question) was only asked at the end of the online teaching. Finally, it is important to note that the survey responses remained anonymous, as was communicated to the students before the survey was administered to them.

### 2.6. Analysis Performed

The aim of this analysis was to obtain the most general possible perspective of the students’ perceptions of online teaching as compared with their perceptions of F2F teaching. To that end, the survey responses were analyzed by type of response.

Firstly, the responses to statements (numbers 1–12 and 14–20) underwent a statistical analysis for an analysis of their average values and confidence intervals. The student evaluations of each course unit (BCU, DCU, and MCU) and teaching methodology, the two main factors of this study, could therefore be compared. However, this type of analysis assumes that there is no interaction between both factors, despite the high likelihood that the perception of the teaching methodology will depend on the type of course [49]. For this reason, the previous analysis was completed with a two-way ANalysis Of VAriance (ANOVA), which gave us detailed insight into which factors were significative for the students in each statement. A significance level of 10%, quite common in this type of analysis, was used [59].

Secondly, the response to question number 13 was considered to be a qualitative variable. Therefore, its analysis was based on the calculation of absolute and relative frequencies. The preparation of sectorial charts offered a simple definition of the main communication channels between the teacher and the students [29].

Finally, open question number 21 was subjected to two types of analysis. Firstly, a qualitative analysis was performed, which provided a broad yet detailed picture of the positive and negative aspects detected by the students [60]. It was based on the assignment of two levels of crossed codes and on continuous review and feedback from the authors. A mixed analysis based on word counting was also conducted, in order to endorse the conclusions obtained through the qualitative analysis. It meant that the most frequently highlighted aspects in the responses could be easily determined [61].

## 3. Results and Discussion

This section presents the results obtained after the analysis of the responses from the 66 students participating in the experiment. The presentation of the results was divided according to the type of analysis (explained in Section 2.6).

### 3.1. Numerical Rating Statements: Average Values and Confidence Intervals

The students rated statements 1–12 and 14–20 in the survey from 1 to 5 on a Likert-type scale (see Section 2.5) both with regard to F2F and with regard to online teaching. These statements addressed the explanation and learning of theoretical and practical concepts, the quality of the teaching material, and the communication with the teacher, as well as their attitude during teaching. The analysis of the valuations to these statements was performed by calculating the average values and the confidence intervals.

#### 3.1.1. Explanation and Learning of Theoretical Concepts: Statements 1–3

**Figure 1:**
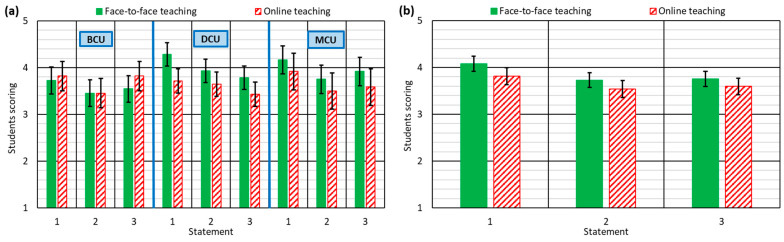
Student responses to statements 1–3: (**a**) for each type of course unit; (**b**) for all course units.

The main conclusion is that the students considered that the theoretical concepts had been properly explained and learnt during both the F2F and the online teaching period, as can be seen from the high ratings that they provided (Figure 1). There are also some other aspects that are worth noting.

As shown in Figure 1a, in both the DCU and the MCU, the students thought that the explanations and the understanding of the theoretical concepts (statements 1 and 3) were better during the F2F teaching. However, the opposite was noted for the BCU: the explanations and understanding of the theory not only never worsened, but even slightly improved during the online teaching. The teachers involved explained that they were aware that the theoretical load of the basic courses is greater [62] and that the students are younger, and less experienced at autonomous learning [47], so that may have led them to prepare the videos/audios in which theoretical concepts were explained more carefully. The novelty (exceptionality) of the situation led to increased dedication to these points of interest and improvements of the traditional F2F explanations.

On the other hand, the students considered that the F2F teaching prepared them better for the practical application of the concepts that they had learnt throughout the course in their professional working life (statement 2), regardless of the course type. Undoubtedly, F2F teaching implies a closer relationship and greater teacher–student interaction, usually presenting example applications, real cases, and even personal experience related to the content of the course unit, bringing the future engineer closer to the professional field [63]. However, the absence of this contact in the online teaching meant that the focus of the teacher was exclusively on the curricular content and the aforementioned type of knowledge was usually omitted [56].

A global analysis of the responses to these statements (Figure 1b) showed that students valued online teaching less than F2F teaching. Nevertheless, the difference in their evaluations was, on average, only 0.3 points, which reflects the solid work of the teachers when preparing the videos/audios that explained the theory. Furthermore, the greater dispersion (width of the confidence intervals) of the responses from the students with regard to online teaching showed lower homogeneity in their evaluations.

#### 3.1.2. Explanation and Learning of Practical Concepts: Statements 4–6

It is widely accepted that the greatest shortcoming of online teaching is the explanation of practical concepts [48]. The teacher cannot check whether students have properly understood the exercises, due to the absence of direct contact and observation of practical exercises [50]. As expected, this situation was reflected in the results of statements 4–6, although, once again, both types of teaching received similar ratings, which reflects the capabilities of the teachers to adapt to online teaching within a very short time.

**Figure 2:**
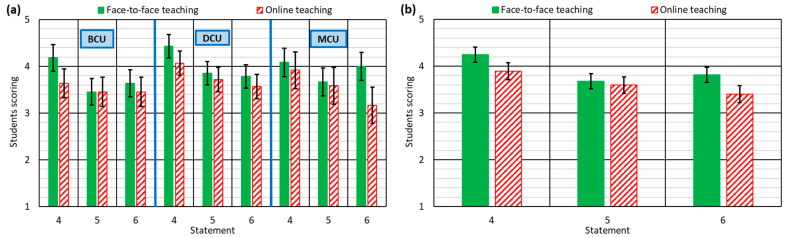
Student responses to statements 4–6: (**a**) for each type of course unit; (**b**) for all course units.

The three dimensions of learning practical concepts (their explanation, statement 4; the student’s ability to apply them in the professional world, statement 5; and their understanding, statement 6) were rated lower by students in online teaching, as shown in Figure 2b. However, two important aspects may be observed.

- The results were worse than for the theoretical concepts, reflected by the lower valuations of statements 4 and 6 at around 0.4 points. It confirms that it is more difficult to achieve a correct explanation and understanding of practical concepts in online teaching.
- Figure 2a shows that the best results in this section were for the DCU, regardless of the type of teaching. These course units generally have a highly practical focus, and it is usual for the teacher to emphasize design concepts [53], an attitude that was maintained during the online teaching, which meant that the DCU obtained the highest score in this section.
- The overall rating of statement 5 in online teaching was only 0.1 points lower than F2F teaching (see Figure 2b). The difference in statement 2 (application of theoretical concepts in the professional field) was 0.2 points. The teachers usually linked the solutions of the exercises to aspects of the professional world in which they are experienced [63]. Although this practice is more difficult in online than in F2F teaching, it was observed that the teachers maintained it more easily than in the explanation of theoretical concepts.

#### 3.1.3. Quality of Teaching Material: Statements 7–9

The preparation of quality teaching material is a fundamental aspect in online teaching, especially if it is taught asynchronously [51]. In this type of teaching, although the teacher provides videos or audios in which the concepts are explained, the doubts that the students may have must be resolved to a great extent by themselves, because the teacher will not be available during the explanation of the concepts [22]. Careful preparation of this material is therefore very important, in the absence of real-time meetings between the teacher and the students during the explanation, so that the students can understand the concepts as they are addressed [52].

**Figure 3:**
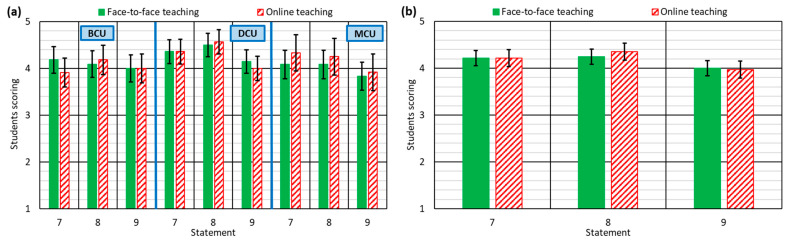
Student responses to statements 7–9: (**a**) for each type of course unit; (**b**) for all course units.

As mentioned, the preparation time of quality notes, presentations, videos, and audios is copious [50]. However, the totally unexpected situation caused by the COVID-19 pandemic between March and June 2020 prompted teachers to adapt from F2F teaching to online teaching very quickly [30]. It meant that they hardly had sufficient time to apply all the teaching material to online teaching. However, this situation was not evident in the perceptions of the students, who considered that the teaching material was prepared for online teaching (statement 7) and was of high quality (statement 8), as the overall ratings of these statements were 4.2 and 4.4 out of 5, respectively (see Figure 3b). In addition, the students reported that the teaching material provided during online teaching was better in all the course units, which again reflects the capability of the teachers to adapt to the situation and to dedicate time to online teaching of the course units, despite any problems of balancing family and professional life [64].

Another problem often observed in online teaching is excessive documentation provided to students [50]. It can sometimes disorient students with regard to the most relevant aspects of the course [48]. In addition, any disorientation might have been exacerbated due to psychological problems such as anxiety [35] and stress [34] during confinement. However, this problem was not noted in this experiment (statement 9). In fact, both F2F and online teaching obtained the same score in this section: 4 points (see Figure 3b). The analysis by groups of courses yielded the same conclusion (Figure 3a). Once again, the teachers were aware of the difficult situation that they themselves and their students had to face, and they provided only the essential material for learning of the course unit concepts.

#### 3.1.4. Teacher’s Availability to Communicate with Students: Statements 10–12

All the aspects addressed in Section 3.1.1, Section 3.1.2, and Section 3.1.3 should be accompanied by optimal communication between the student and the teacher. In asynchronous online teaching, it is essential that students feel that the teacher cares about them and actively participates in learning [24], as it increases motivation during the course [51]. However, it also implies that the teacher must be available on a very flexible schedule to answer the doubts raised as quickly as possible, so as not to hinder the pace of student learning [25].

**Figure 4:**
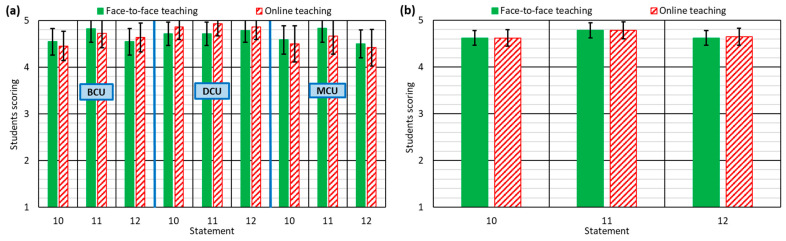
Student responses to statements 10–12: (**a**) for each type of course unit; (**b**) for all course units.

Communication between teacher and students was a major concern for the authors of this research work, as it was thought that the consequences of lockdown and the consequent change in lifestyle [3] might imply difficulties for some teachers when addressing the questions posed by students. However, the valuation of the students pointed to the solid work performed by the teachers, as the students rated ease of contacting the teacher (statement 10), the willingness of teachers to answer all kinds of questions (statement 11), and the speed with which the teacher answered their questions (statement 12) with more than 4.5 points out of 5 (see Figure 4b). In addition, all these aspects were valued slightly better in online than in F2F teaching, which reflects the commitment of the teachers involved, despite the difficult circumstances.

#### 3.1.5. Students’ Assessment of Teachers’ Attitudes during Teaching: Statements 14–16

Statements 14–16 evaluated the attitudes of teachers during each course unit. Students were asked whether they felt the teachers were concerned about their understanding of the concepts (statement 14) and whether the teachers had checked their understanding (statement 15). These are two common shortcomings in asynchronous online teaching [52], mainly due to the absence of direct contact between the teacher and the student [22]. In online teaching, the teacher must continuously monitor student learning to adapt it to their needs as they arise [23]. This can be done through questions by email or through meetings using online tools (Skype, Microsoft Teams …) [29].

**Figure 5:**
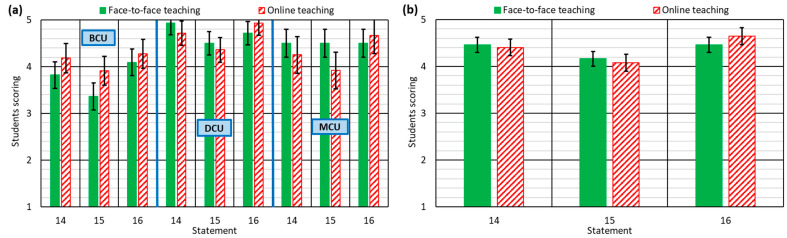
Student responses to statements 14–16: (**a**) for each type of course unit; (**b**) for all course units.

The average valuation of these aspects in online teaching was almost the same as in F2F teaching when all the course units were jointly analyzed, as shown in Figure 5b. However, the analysis by groups of course units showed some relevant aspects (Figure 5a). 

Teaching attitudes of the DCU and MCU were more valued than in the BCU. As explained in Section 3.1.1, the students of BCU are younger (around 2 years younger on BCU on average than on DCU and MCU) and at the start of their university career, when their knowledge base is weaker [62]. It implies that they tend to need closer monitoring, as they have less capacity for autonomous learning, which makes the valuation of this aspect lower than in more advanced courses [65].

In contrast, while teaching attitudes were valued more negatively in the online teaching of the DCU and the MCU, the valuation of the BCU was 0.4 points higher for online teaching, reflecting the concern of the BCU teachers to provide further support to students for proper understanding of the concepts [65], so they improved their performance online [63].

Furthermore, students were asked whether the teacher was sympathetic towards handing in project assignments late in the day (statement 16). This last question was fundamentally asked due to the social situation in which online teaching was performed [2]. Both the family and the domestic situation of each student might vary during the confinement [33], and the authors of this experiment considered that it was a highly relevant aspect that had to be studied. The results showed that teachers increased their understanding during online teaching, regardless of the type of subject (Figure 5a). It proves that teachers were aware of the difficulty of working in lockdown and quickly and successfully adapted to it, showing high levels of empathy towards their students.

#### 3.1.6. Overall Perception of the Course: Statements 17–20

All the aspects discussed in the previous sections influenced the global perspective of the students towards the course units [47]. The purpose of the last questions of the survey was to establish whether the students considered that the course had been difficult to understand (statement 17) or to pass (statement 18), whether they thought they had learned (statement 19), and whether they had a high workload (statement 20). These direct questions (see Section 2.5) were asked to elicit replies from the students that were focused on the specific aspects that the authors of this research work wished to analyze [55].

**Figure 6:**
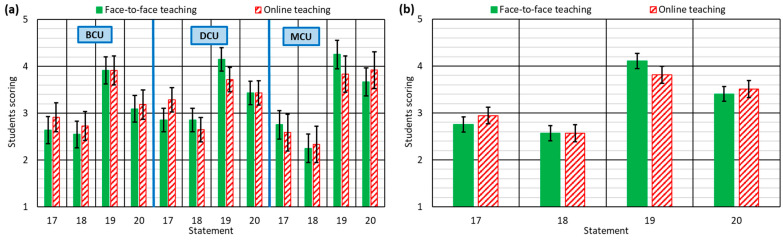
Student responses to statements 17–20: (**a**) for each type of course unit; (**b**) for all course units.

There were no major differences between both teaching methodologies (see Figure 6). However, some aspects should be highlighted:

- Firstly, as expected [13], the students considered that the course unit concepts were more difficult to understand in online teaching. The lack of familiarity with online teaching among both students and teachers may mean that globally the concepts were slightly less well explained [22]. It also meant that students thought they had learned less through online teaching [50]. However, the difference between both types of teaching was very small, due to the aspects highlighted in previous sections.
- Various studies have shown that if a course is graded in online mode in the same way as in F2F, the online grades are clearly lower [66]. It is therefore essential that grading methods are adjusted to the type of teaching [58]. For these reasons, the analysis of course-unit difficulty yielded surprising results, because the students considered it similar in both types of teaching. In addition, the DCU taught online were considered easier to pass. When asked, teachers indicated that the grading method had to be adapted in the middle of the term, after receiving some guidelines from the University for the conversion to virtual teaching and, especially, to fully online assessment. Moreover, some common strategies were detected: the relevance of the exam in the final grade was reduced in all courses, and the weight of the project assignments was increased. As commented upon with regard to other aspects, the teachers also showed a great capability to adapt the grading method of the course unit to the existing situation. It is important to point out that in no way could the design of the experiment be said to condition the grading of the courses (see Section 2.3).
- In line with the above, the students never indicated that the workload varied much between both types of teaching, although it was slightly higher in online teaching. In this type of teaching, the time spent memorizing content in the absence of classroom learning processes is greater than in F2F teaching [30]. However, the relevance of the exam in the final grade was decreased in all the courses, which reduced the need for memorization. In this way, students spent more time on project assignments, work that they considered more enjoyable and that may have given them the impression of a lighter workload.

The general analysis of these observations suggested that the teachers, despite the short time available and the unexpected outbreak of the COVID-19 pandemic, showed great adaptability, in so far as they taught their course units with similar results to those obtained in F2F teaching.

### 3.2. Numerical Rating Statements: Effect of the Factors, Two-Way ANOVA

The descriptive analysis presented in the previous sections has covered any slight variations of student opinions. However, all the points addressed in the survey can be globally analyzed using the two-way ANOVA. This statistical procedure tells us whether each factor (type of course and teaching) has a significant influence on student perceptions of each aspect that is analyzed [59]. Moreover, the interaction between the factors can be considered in this type of analysis, in such a way that the significance of each factor is analyzed according to the effect of the other [67]. In short, it is a statistical procedure that generates a faithful representation of reality [68]. In this study, the two-way ANOVA was performed to a confidence level of 90% (significance level of 10%). The *p*-value obtained for each factor in each survey question is shown in Table 2, as well as the homogeneous groups (factor values between which there are no significant differences).

On the one hand, it may be observed that the type of course unit had a significant effect upon the teachers’ attitudes towards their teaching and student perceptions of the teacher’s concern to impart an understanding of the concepts to the students (statements 14–16) and on the amount of work required (statement 20). All these points were not controlled in the design of the experiment (see Section 2.2), but were rather closely related to the personality traits and behavior of each teacher [43]. It is clear that each teacher has an individual way of behaving and sharing in the classroom that cannot be fairly assessed through survey questions alone [49]. In addition, the homogeneous groups showed that there was no significant difference between the DCU and the MCU regarding these aspects. The superior autonomous learning of the students, and their greater knowledge of the basic concepts when following these courses, led the teachers to adopt significantly different attitudes compared to the BCU, a common situation also remarked upon in another study [65]. It may therefore be affirmed that the type of subject matter had no influence over the students’ perception of the controllable aspects of the experiment.

The type of teaching significantly influenced two different aspects: the understanding of the practical concepts and the students’ perceptions of whether they had learned during the course unit. These two concepts are usually problematic in online teaching [56]. On the one hand, the explanation of the practical aspects is not done in direct contact with students, so they have no possibility of interacting with the teacher in real time [40]. Although this problem is slightly reduced if the explanation of the practical concepts is performed through online meetings where students can raise any doubts more easily [69], it has been demonstrated in various studies that online meetings and the understanding of practical concepts are less effective than personal meetings [57]. On the other hand, it is common for students to consider that their learning during online teaching is less effective, although in some cases this opinion does not reflect reality [50]. Another reason is the lack of direct contact with other students, so that they cannot compare their learning with their peers [57]. The lack of direct contact with the teacher and, therefore, fewer explanations from the teacher of personal experience that is shared with students also favors this perception [43]. It may therefore be observed that online teaching only presented significant differences with F2F teaching in those aspects that are somehow “inevitable”. It all reflected the great effort of the teachers to adapt to online teaching and, for the authors of this study, was somewhat surprising.

### 3.3. Channels of Communication between the Teacher and the Students

**Figure 7:**
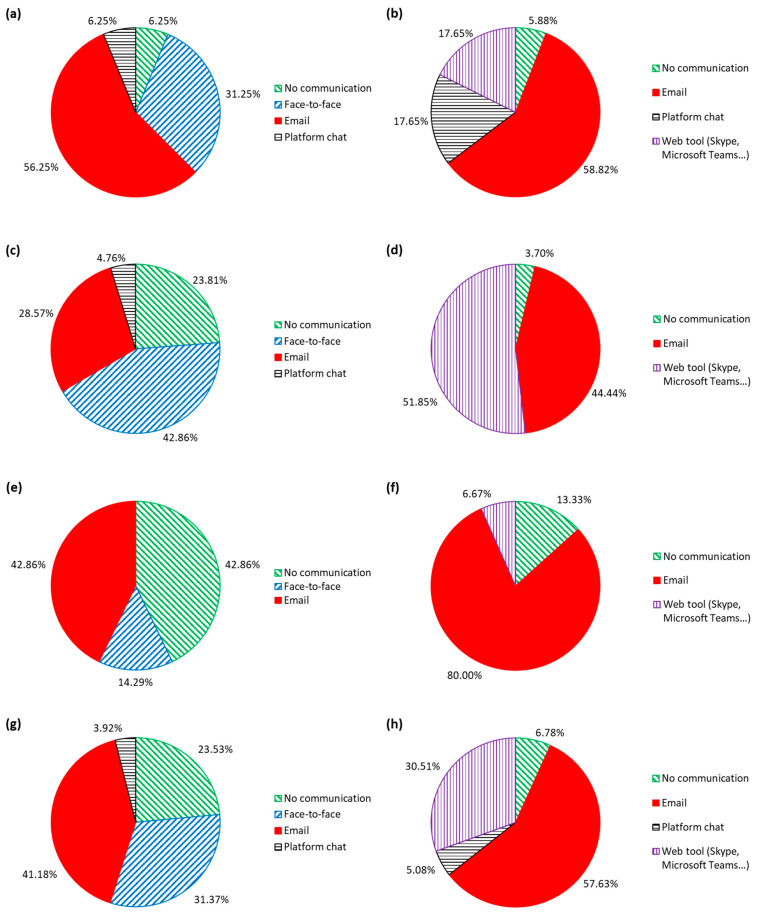
Communication channels of students with teachers: (**a**) BCU, F2F teaching; (**b**) BCU, online teaching; (**c**) DCU, F2F teaching; (**d**) DCU, online teaching; (**e**) MCU, F2F teaching; (**f**) MCU, online teaching; (**g**) All course units together, F2F teaching; (**h**) All course units together, online teaching.

The aim of question 13 was to ascertain the main communication channels between the teacher and the students. Five different alternatives were identified from the students’ responses: the student asked the teacher nothing (no communication), F2F, by email, through a chat on the University of Burgos web platform for teaching (MOODLE), or via a web tool such as Skype or Microsoft Teams. As indicated in Section 2.2, in the design of the experiment, teachers were asked to answer the questions sent by email as quickly as possible and to set a tutorial schedule for the resolution of doubts through an online tool. Nevertheless, the several communication channels offered by the teachers is further proof of their optimal adaptation to online teaching within a short period of time. The relative frequencies are shown in Figure 7.

If the results are globally analyzed (Figure 7g,h), it can be seen that email was the tool that students used most (approximately 4 out of every 10 students) to communicate with the teacher during F2F teaching, a result that was in line with the digitization of all fields of society, including education [70]. It is nowadays easier for students to communicate with the teacher by email than in person [48], which was the second most frequently used option (3 out of every 10 students). In online teaching, the use of email increased significantly and was used by almost 6 out of 10 students. The teacher can give real-time attention to students through web tools, so those students who communicated in person with the teacher probably used them (3 out of 10) [69]. On the other hand, while 24% of the students raised no doubts with the teacher in F2F teaching, only 7% raised no doubts with the online methodology, which clearly shows that the lack of direct support from the teacher during the online teaching provoked a higher number of doubts among the students [47]. The use of the teaching platform as a communication channel between the students and the teacher was practically non-existent in both types of teaching.

The following global trends were also individually observed in each type of course unit, although there were notable differences between them, mainly regarding the tool type used by students.

- On the one hand, the DCU contained the most engineering design-related concepts that have to be explained [47]. This type of course unit therefore has aspects that are difficult for students to understand [65], which means that F2F communication is very common (according to Figure 7c, 4 out of every 10 students used it) during F2F teaching. In turn, web tools were the most frequently used communication channel among those students (5 out of every 10 students) during online teaching, because it is the closest to F2F communication that can be used over a distance [57]. The use of email also increased (from 3 to 4 out of every 10 students), while the number of students who presented no doubts was practically negligible.
- On the other hand, the teacher’s personal preferences also influenced the communication channel in use. The BCU was the only course unit in which the chats available on the teacher support platform were used by students (see Figure 7a,b), meanwhile the increased use of email, from 4 to 8 out of every 10 students, was very notable in the MCU (see Figure 7e,f). In view of this situation, all the teachers who participated in the experiment were asked if they had promoted the use of some specific communication channel. The BCU teachers indicated that they had encouraged students to communicate via the MOODLE platform chat option. In their opinion, it was similar to email as a means of communication, but they could control the resolution of doubts in a simpler and more effective way, as the finding of other studies have also shown [71]. Furthermore, MCU teachers indicated that they had promoted the use of email because it allowed them to better reconcile their teaching activity with family life.

### 3.4. Global Opinion of Students

Despite the many different aspects addressed in the survey, a final open question in the survey at the end of the online teaching was considered relevant (see Section 2.5). In their responses, students could freely express their opinions and relevant aspects not specifically addressed in the survey might be detected [60]. The answers provided by the students were analyzed both qualitatively and in a mixed way.

#### 3.4.1. Qualitative Analysis

The qualitative analysis revealed some interesting aspects regarding online teaching and its comparison with F2F methodology.

Firstly, the students referred to the greater difficulty with their understanding of practical concepts during online teaching, which was mainly due to the absence of direct contact with the teacher. Some of them also stated that the lack of contact with the other students made learning difficult at some point, since discussing the concepts with peers meant different points of view could be compared and a better overall understanding of the course achieved.

*“I believe that the main disadvantage of online teaching has been the difficulty with understanding the practical exercises.”*

(DCU)

*“The communication with the teacher is not as frequent and easy in online teaching as during F2F teaching. In F2F teaching you can ask the teacher about your doubts while they are explaining the exercise.”*

(BCU)

*“I think that the contact with my classmates is beneficial to know their point of view and understand the concepts better.”*

(DCU)

*“I think that in F2F teaching you get a better overall learning experience.”*

(MCU)

However, students also highlighted another point that was not addressed in the other survey questions: a routine is established in F2F teaching, whereas in online teaching (especially in asynchronous mode) the student has to decide when to study. This control over study hours is mentioned in the literature as a key point that guarantees successful study for an online university qualification [24]. The answers showed that some students considered autonomous control over study hours as a positive aspect that gave them greater freedom to study the course unit. However, others pointed out that defining a specific time to work was very difficult, a situation that was worsened by the confinement. The ease of adapting to this autonomous work depended on two aspects: on the one hand, on the personality of each student, which is the aspect that normally conditions this feature [27] and, on the other hand, on the family and work situation caused by the COVID-19 pandemic, which notably hindered this adaptation [24].

*“F2F teaching forces you to follow a study routine that is beneficial for keeping the course up to date.”*

(MCU)

*“I liked videos explaining the concepts that the teacher provided. In this way, I have been able to follow the course as I wanted and study when it was most convenient for me.”*

(DCU)

Finally, the students also stressed the importance of the teacher’s role in all aspects of their learning process: the amount of work required, the explanation of concepts, the material provided and, fundamentally, the monitoring of students and the resolution of doubts. It shows the importance of the teacher in the whole learning process and that the success of teaching in this exceptional situation has largely depended on their attitude and their working methods, as has also been demonstrated in other teaching methodologies [47]. In general, the students were satisfied with the work performed by the teacher.

*“The teacher has positively adapted the course to the confinement situation. He has sent us weekly work that is not excessive and that has meant we can stay up to date with the course unit without too much stress.”*

(BCU)

*“I would like to thank the flexibility of the teacher when responding to our doubts. She has constantly helped us.”*

(DCU)

*“I am happy with the teacher and with the way she has managed the course during this lockdown period. Her monitoring during the online teaching has also been very useful.”*

(MCU)

*“The teacher has always been attentive to the students and their doubts.”*

(DCU)

#### 3.4.2. Mixed Analysis

**Figure 8:**
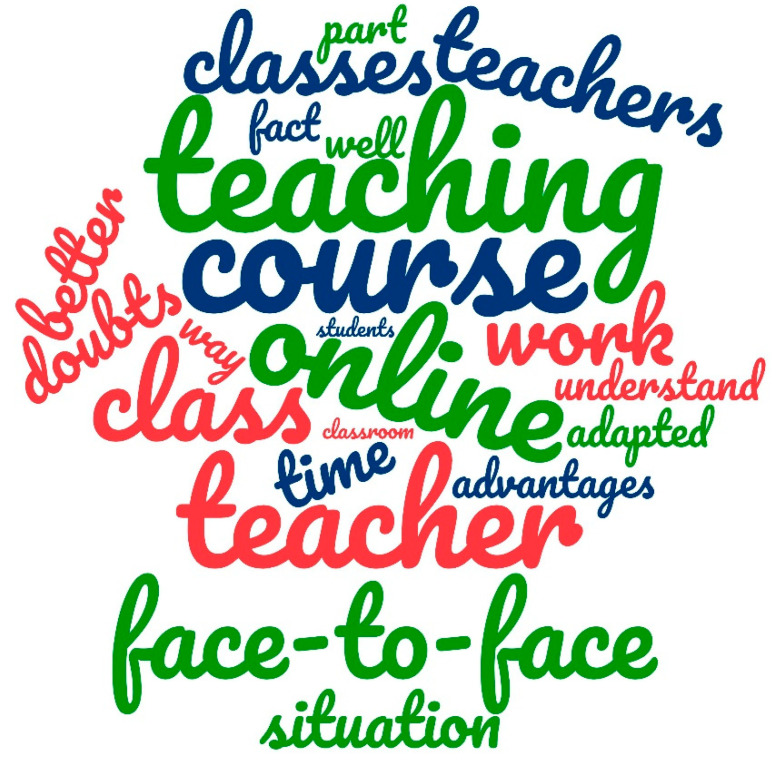
Word cloud of student responses to question 21.

The mixed analysis of the answers provided by the students was based on word counting. This analysis generated the word cloud shown in Figure 8. It can be seen that the students repeated the words “course”, “teaching”, “online”, “F2F” and “teacher” more than any others. The first four words are closely related to the subject matter addressed in the survey, the analysis of which is irrelevant, as indicated in other similar studies [55]. However, the word “teacher” is not directly related to the situation under study and reinforces the abovementioned aspect: the great importance that students attributed to the teacher in both types of teaching, but with special emphasis on online teaching. The participating teachers had never previously conducted this type of teaching and the opinions of the students showed how successfully they were able to adapt to it.

### 3.5. Limitations of the Study

All the results presented throughout this section were in reference to the experiences of the online teaching among 66 engineering students during the lockdown caused by the COVID-19 pandemic on 5 different engineering course units (one BCU, two DCU, and two MCU). It is therefore necessary to highlight two limitations of this study:

- On the one hand, although the types of course units under analysis were the most common, the large number of existing engineering careers meant that some types of course units were not studied in this research work [49]. Therefore, the results of this study should not be considered valid for all course units, as a detailed study may be necessary in some of them.
- On the other, only the students’ opinions were analyzed. The teachers’ reflections on teaching engineering during the lockdown due to the pandemic were not evaluated. Doing so evaluated whether the teachers were able to adapt correctly to online teaching, but not how they did it [50].

## 4. Conclusions

Throughout this study, the perceptions of engineering students on the quality of online teaching received during the confinement due to the COVID-19 pandemic have been evaluated. Moreover, those perceptions have also been compared with the face-to-face (F2F) teaching conducted at the start of the course before the pandemic. Several aspects have been discussed: the learning and explanation of the theoretical and practical concepts, the quality of the teaching material, the ease of teacher–student communication, and the teachers’ attitudes. Finally, the students’ global vision of the course has also been analyzed.

The results have shown that the students’ assessments of the quality of both F2F and online teaching were very similar and greatly depended on the work of the teacher. In fact, rather than perceiving a worsening of various aspects of teaching quality due to the abrupt adaptation to online teaching, in some cases they even stated that the quality of teaching had improved:

- Students considered that the explanations of theoretical concepts were more successful during F2F teaching, except in the basic course units taught in the first years of engineering degrees. The reduced learning autonomy of the recently enrolled students increased the concerns over the explanation of these types of concepts among the teachers [47].
- Despite the need for teachers to balance family and professional life [7], the students indicated that the teaching material provided was prepared in detail, and was adequately adapted to online teaching. In addition, the documentation was not in excess, which is a common problem when teaching online [48].
- The attitude of the teachers towards the students was attentive, expressing constant concern for their learning. The basic course units experienced a notable improvement in this aspect, because students in the first years of their careers were more dependent on the teacher in their learning [62].
- Course unit grades were not influenced by the abrupt change in the teaching modality, due to the way that the teachers had adapted the evaluation system. An online course unit that is graded in the same way as a F2F course will usually result in significantly lower grades [66].

These results have demonstrated the solid training of engineering teachers in a twofold manner: firstly, their training for teaching tasks, based on their knowledge information technology that has facilitated their abrupt adaptation to a new type of teaching in a very successful way [53]; secondly, their social and human skills, in so far as they were aware of the different (in some cases, quite complicated) family conditions of their students during the confinement when adapting to online teaching [43]. In this way, they defined an amount of work, a level of communication with the student, and grading requirements that in no case were negatively evaluated.

However, a significantly negative influence of online teaching was also detected: the understanding of practical concepts and the perception of learning during the course. These common shortcomings of online teaching are due to the lack of direct teacher–student contact and among students themselves [50] that new technologies and teaching methodologies should seek to improve. Furthermore, it was noted that the perception of learning during the course depended on students’ personality, which conditioned, for example, their ability to self-regulate their learning by defining their own study schedules [27]. In relation to the type of (basic, design, and management) course unit, differences were associated with the nature of the concepts taught in each of them [49], but they were only statistically significant with regard to aspects that depended on the personality traits of the teacher.

Regarding the communication channels between the teacher and the students, it was observed that, in general, during both online and F2F teaching, the same trend was maintained: the main communication tool between teacher and student was email. However, the use of web tools (Skype, Microsoft Teams …) increased when their use was necessary for the explanation of practical concepts. In addition, specific differences were detected according to the preferences of each teacher, which led to the promotion of the use of one or another communication channel [71].

Based on the opinions of the students themselves, it can be concluded that the training of the future engineers during the confinement period was adequate and that there were no notably negative differences with the F2F teaching that they have traditionally received. Their training is therefore preparing them well for their future professional activity in their roles as engineers contributing to the development of society. Furthermore, if restrictive situations are to be imposed again in the future, the engineering teachers will be even better prepared than before to offer online classes that respond to the teaching–learning needs of their students.

## Figures and Tables

**Table 1:** Mean age of students for each course unit.

Course	Number of Students	Mean Age of Students
Business Economics II	19	20.09 ± 1.04
**Basic Course Units (BCU)**	**19**	**20.09 ± 1.04**
Construction & Agroalimentary Building	17	21.70 ± 2.41
Engineering of Green Spaces	9	22.75 ± 1.50
**Design Course Units (DCU)**	**26**	**22.00 ± 2.18**
Project and Construction Management	14	20.88 ± 0.99
Engineering Projects	7	23.75 ± 1.59
**Management Course Units (MCU)**	**21**	**22.14 ± 2.14**

mean value for each course-unit type in bold.

**Table 2:** Results of two-way ANOVA.

Statement	*p*-Value. Factor: Course Type (BCU/DCU/MCU)	Homogeneous Groups. Factor: Course Type (BCU/DCU/MCU)	*p*-Value. Factor: Teaching Type (F2F/Online)
1	0.1625	-	0.4956
2	0.2459	-	0.2409
3	0.7707	-	0.3305
4	0.3530	-	0.1162
5	0.1198	-	0.5329
6	0.8408	-	**0.0395**
7	0.3847	-	1.0000
8	0.1033	-	0.5227
9	0.6482	-	0.8789
10	0.3095	-	1.0000
11	0.8764	-	1.0000
12	0.1122	-	0.8531
14	**0.0002**	BCU and MCU	0.7221
15	**0.0107**	DCU and MCU	0.7023
16	**0.0004**	DCU and MCU	0.1376
17	0.3970	-	0.4663
18	0.1495	-	1.0000
19	0.6904	-	**0.0289**
20	**0.0124**	DCU and MCU	0.5256

significant *p*-values in bold.

## Data Availability

The data presented in this study are available on request from the corresponding author. The data are not publicly available due to the need to maintain the participants’ anonymity.
